# The Social Cost of Ozone-Related Mortality Impacts From Methane Emissions

**DOI:** 10.1029/2023ef003853

**Published:** 2023-09

**Authors:** Erin E. McDuffie, Marcus C. Sarofim, William Raich, Melanie Jackson, Henry Roman, Karl Seltzer, Barron H. Henderson, Drew T. Shindell, Mei Collins, Jim Anderton, Sarah Barr, Neal Fann

**Affiliations:** 1 Office of Atmospheric Protection, Climate Change Division, U.S. Environmental Protection Agency, Washington, DC, USA,; 2 Industrial Economics, Incorporated, Cambridge, MA, USA,; 3 Office of Air Quality Planning and Standards, Air Quality Assessment Division, U.S. Environmental Protection Agency, Research Triangle Park, NC, USA,; 4 Nicholas School of the Environment, Duke University, Durham, NC, USA,; 5 Office of Air Quality Planning and Standards, Health and Environmental Impacts Division, U.S. Environmental Protection Agency, Research Triangle Park, NC, USA

## Abstract

Atmospheric methane directly affects surface temperatures and indirectly affects ozone, impacting human welfare, the economy, and environment. The social cost of methane (SC-CH_4_) metric estimates the costs associated with an additional marginal metric ton of emissions. Current SC-CH_4_ estimates do not consider the indirect impacts associated with ozone production from changes in methane. We use global model simulations and a new BenMAP webtool to estimate respiratory-related deaths associated with increases in ozone from a pulse of methane emissions in 2020. By using an approach consistent with the current SC-CH_4_ framework, we monetize and discount annual damages back to present day values. We estimate that the methane-ozone mechanism is attributable to 760 (95% CI: 330–1200) respiratory-related deaths per million metric tons of methane globally, for a global net present damage of $1800/mT (95% CI: $760–$2800/Mt CH_4_; 2% Ramsey discount rate); this would double the current SC-CH_4_ if included. These physical impacts are consistent with recent studies, but comparing direct costs is challenging. Economic damages are sensitive to uncertainties in the exposure and health risks associated with tropospheric ozone, assumptions about future projections of NO_x_ emissions, socioeconomic conditions, and mortality rates, monetization parameters, and other factors. Our estimates are highly sensitive to uncertainties in ozone health risks. We also develop a reduced form model to test sensitivities to other parameters. The reduced form tool runs with a user-supplied emissions pulse, as well as socioeconomic and precursor projections, enabling future integration of the methane-ozone mechanism into the SC-CH_4_ modeling framework.

## Plain Language Summary

The social cost of methane is used to assess the costs and benefits associated with emissions mitigation in U.S. regulations, in addition to other decision-making applications. The current social cost of methane used by the U.S. Government is $1500/metric ton of methane emissions. This estimate does not include damages related to deaths associated with changes in exposure to background ozone, resulting from increases in atmospheric methane. Using an approach consistent with the social cost of methane framework, we estimate that damages from the methane-ozone mechanism are $1800/metric ton, which, if included, would double the current social cost of methane. These costs have uncertainties related to the health risks associated with exposure to ozone, assumptions about future NO_x_ emissions, choice of discount rates, and other factors. We also develop a reduced form model that allows rapid estimation of many of these sensitivities and enables consideration of this mechanism in the social cost methodology.

## 1. Introduction

Methane is emitted from a variety of natural and anthropogenic sources (e.g., agriculture, wetlands, oil and gas activities, coal mining, etc.) and is the second most important greenhouse gas (GHG) behind carbon dioxide (CO_2_), having contributed to roughly half a degree of present-day warming (and ~1/3 of total GHG-induced warming). Methane, however, has a shorter atmospheric lifetime than CO_2_ (a perturbation lifetime of ~12 years, contrasting with CO_2_’s lifetime of centuries to millennia), such that reductions in global methane emissions can lead to reductions in atmospheric concentrations in only a matter of years (IPCC, 2021). Recently, under the Global Methane Pledge, over 150 participants agreed to reduce global methane emissions by 30% by 2030 relative to 2020 levels, which has been projected to decrease mean midcentury global surface warming by 0.2°C (CCAC Secretariat, 2021). The social cost of methane (SC-CH_4_) (Errickson et al., 2021; Marten & Newbold, 2012; Shindell et al., 2017) has been used to value these and other types of direct climate benefits associated with marginal methane emission changes, most recently valued at roughly $1500 (2020$, 2020 emissions, 3% economic discount rate) (Interagency Working Group on Social Cost of Greenhouse Gases (IWG), 2021) or $1600 (2020$, 2020 emissions, 2% Ramsey discounting rate) (EPA, 2022) per metric ton of methane (mT CH_4_). These estimates include damages to human health, agriculture, energy, and labor associated with projected increases in surface temperatures and other climate responses to changes in atmospheric methane concentrations.

In addition to these direct impacts, methane also contributes to the chemical formation of tropospheric ozone. Ozone in the troposphere is a GHG and air pollutant, responsible for over 11% of chronic respiratory deaths attributable to outdoor air pollution worldwide each year (GBD, 2019 Risk Factor Collaborators, 2020), as well as global agricultural crop damages of over $34 billion (in 2010 in 2015$, Sampedro et al., 2020). Ozone formation in the troposphere occurs from the reaction of volatile organic compounds (VOCs) or carbon monoxide with nitrogen oxides (NO_x_ = NO + NO_2_) in the presence of sunlight. Methane’s 12-year lifetime is much longer than the hour-to-week lifetimes of most other organic ozone precursors. Therefore, methane becomes relatively well-mixed in the atmosphere and ozone production from methane’s oxidation contributes to “background” levels of ozone, rather than localized production. While localized ozone production is an important consideration for regional air pollution mitigation policies, the United States Environmental Protection Agency (EPA) has long recognized that methane mitigation is a poor candidate for addressing local air quality problems. Since 1977, the EPA has exempted methane from the definition of “volatile organic compound” on the grounds that methane has “negligible photochemical reactivity.” (40 CFR 51.100(s)(1), 2023); “Recommended Policy on Control of Volatile Organic Compounds,” (42 Fed. Reg. 35314, 8 July 1977). As a result, the EPA does not regulate methane as part of its programs to implement the national ambient air quality standards for ozone. The health effects of ozone, however, are determined by total tropospheric concentrations, which are a combination of local/regional ozone production and the global background. In contrast to localized ozone, changes in background ozone concentrations occur on time scales similar to methane’s lifetime (e.g., ~12 years), are relatively insensitive to specific locations where emission changes occur, and have been shown to respond linearly to changes in methane (e.g., West et al., 2006). These large multi-year and global scale impacts make this methane-ozone mechanism a good candidate for the social cost of carbon framework.

Previous studies (Anenberg et al., 2012; Sarofim et al., 2017; Shindell et al., 2012; West et al., 2006) have leveraged the relative uniformity in the ozone response to methane changes to estimate global health damages per metric ton of methane. These estimates are generally of the same magnitude as the climate damages from the social cost of methane. Many of these and other studies have also estimated methane-ozone damages from other effects, such as short-term health impacts (e.g., asthma-related hospital visits) and agricultural crop losses, which can also account for a sizable fraction of current SC-CH_4_ estimates (e.g., Sampedro et al., 2023; UNEP/CCAC, 2021). Current SC-CH_4_ values only account for climate-driven damages from methane emissions (including radiative forcing changes from methane-produced tropospheric ozone), indicating that incorporating the additional global health and monetary benefits associated with long-term exposure to methane-produced ozone would be an important modification to the social cost framework.

Most recently, the UN Environmental Program and Climate and Clean Air Coalition (UNEP/CCAC) published the Global Methane Assessment report (UNEP/CCAC, 2021), which included estimates of the physical and economic impacts to global mortality, morbidity, labor productivity, and agricultural yields attributable to ozone produced from methane oxidation. Of these categories, the greatest physical and economic impacts were from mortality associated with respiratory and cardiovascular diseases attributable to long-term (i.e., chronic) exposure to methane-produced ozone, which led to over 1,400 deaths per million metric tons of methane. UNEP/CCAC results were derived from a series of global composition-climate model (GCM) simulations in which methane mixing ratios were reduced by 556 ppbv (50% of the global anthropogenic increase relative to pre-industrial levels) and compared to base simulations. Consistent with previous modeling studies (e.g., West et al., 2006), these simulations showed that background ozone levels respond linearly to atmospheric methane changes of at least ±50% of the total anthropogenic contribution and are only mildly sensitive to changes in other precursor emissions (UNEP/CCAC, 2021). From these simulations, changes in regional ozone levels per mT of global CH_4_ emissions can be calculated in a manner that can be incorporated into the social cost framework, enabling the consideration of additional ozone-health impacts from methane to be considered in cost-benefit analyses.

This analysis is designed to apply five principles that leverage and combine key advances from previous studies. First, to better align with the social cost framework, we assess the integrated impact of a marginal methane emissions pulse on ozone mixing ratios through the end of the century, rather than ozone changes associated with instantaneous and sustained emission reductions. This approach is similar to Sarofim et al. (2017). Second, we use changes in summertime maximum-daily 8-hr average (MDA8) ozone mixing ratios associated with methane concentration perturbations, as derived from the recent UNEP/CCAC simulations. The use of these gridded response maps allows us to capture spatial differences in the magnitude of ozone’s methane response, resulting from regional differences in precursor emissions and chemical production regimes. Third, we use a global instance of the Environmental Benefits Mapping and Analysis Program (BenMAP) webtool to estimate the chronic respiratory-related mortality impacts attributable to perturbed ozone mixing ratios. This is the first application of global BenMAP, which uses the most-recently developed ozone exposure-mortality response function from the 2019 Global Burden of Disease (GBD) project, as well as updated projections of population and background mortality statistics. Fourth, we use estimates for the value of a statistical life (VSL) to monetize the costs associated with annual methane-ozone attributable deaths through the end of the century and integrate and discount these damages in a manner consistent with the most recent SC-GHG framework (Rennert et al., 2022a) to derive a net present damage value per mT of methane emissions. This approach is consistent with the methodology used for U.S. government calculations of the SC-CH_4_ and with the health valuations used for air quality analyses by the U.S. EPA (though the assumptions necessary for global and multi-year lifetimes differ from those acceptable for local air quality analyses). Lastly, we describe the development of a new reduced form tool that uses these results to quantify ozone-related mortality changes associated with projections of perturbed methane emissions for any country and under any emission or socioeconomic scenario. This reduced form model allows for the integration of indirect methane-ozone mortality impacts into the social cost framework and provides insight into the sensitivity of this mechanism to uncertain parameters.

## 2. Materials and Methods

**Figure 1.**
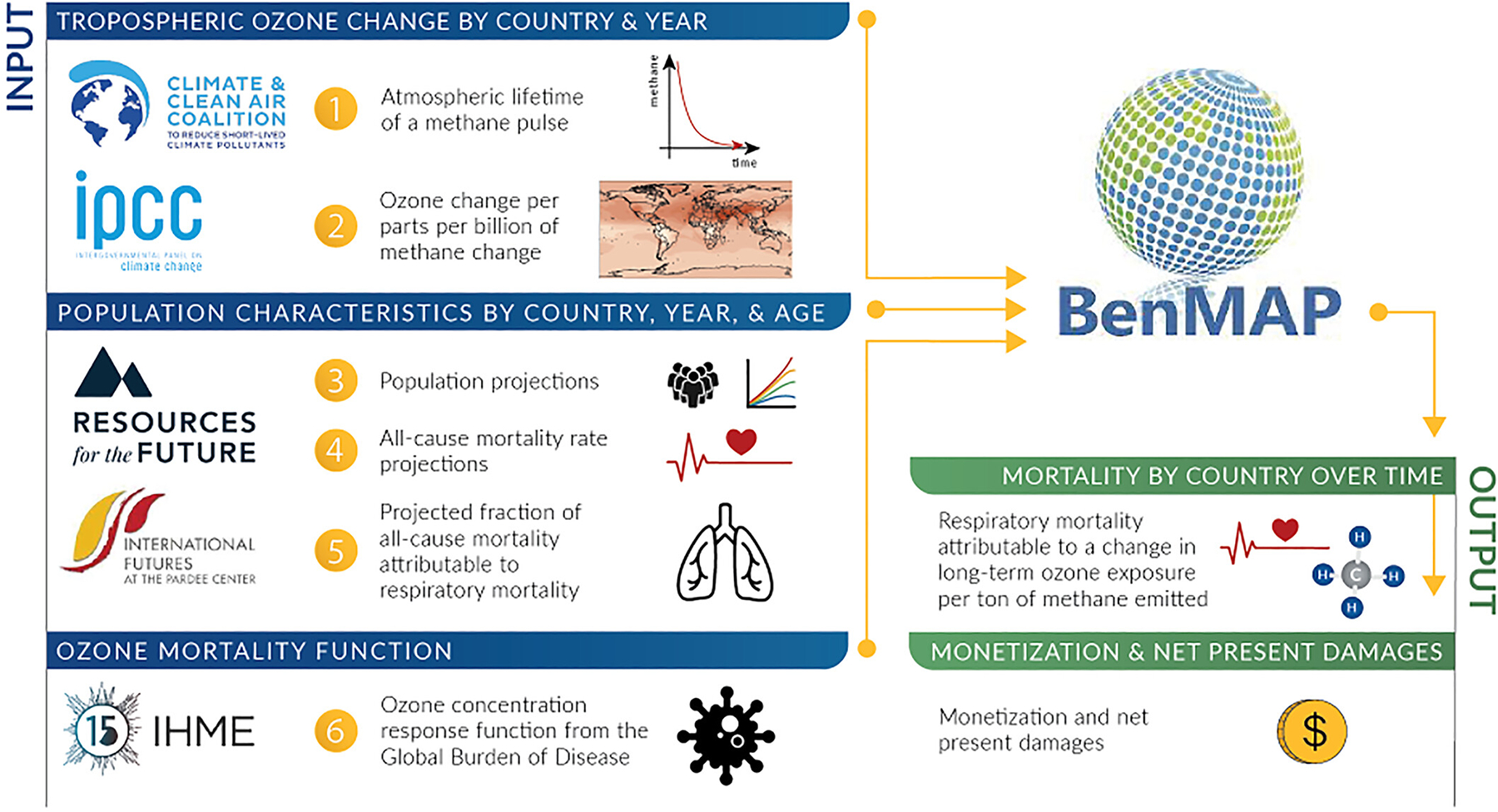
Schematic of analysis workflow. Logos for individual groups and initiatives are used for illustrative purposes only and do not represent endorsement.

This analysis uses a multi-step approach outlined in Figure 1 to calculate the monetary value of additional respiratory-related deaths through the end of the century from ozone exposure associated with emitting a metric ton of methane in 2020. Briefly, global methane-ozone response maps (i.e., O_3_ pptv/CH_4_ ppbv) are used to estimate the annual change in ozone expected from a marginal pulse of methane emissions in the year 2020. The resulting ozone maps are then used as input with projected population characteristics and background mortality in a new application of the global BenMAP webtool to estimate the attributable respiratory health impacts. Annual deaths in each country are then monetized, discounted back to present day values, and aggregated over the century to produce an estimate of the global net present damages associated with ozone from a ton of methane emissions in 2020. This approach enables the estimation of ozone-related mortality benefits associated with methane emission mitigation policies and is well suited to regulatory analysis. All monetary values presented in this analysis are in 2020 U.S. dollars. The following sections provide details about each of the methodological steps and underlying data.

### 2.1. Tropospheric Ozone Change From a Pulse of Methane

We first estimate the annual change in global atmospheric methane mixing ratios over the 21st century, in response to a 275 million metric ton (or ~100 ppbv) methane emissions pulse in the year 2020 (Figure S1, left in Supporting Information S1). For this calculation we use the atmospheric perturbation lifetime of methane of 11.8 years from the IPCC AR6 (Szopa et al., 2021) (Figure 1(1)) and the methane mass to mixing ratio (Tg/ppbv) conversation factor from Prather et al. (2012) (Section S1 in Supporting Information S1).

To estimate the annual amount of ozone produced from this pulse, we then leverage global maps of changes in tropospheric ozone resulting from atmospheric methane changes, previously simulated as part of the UNEP/CCAC Global Methane Assessment (UNEP/CCAC, 2021) (Figure 1(2)). As described in the UNEP/CCAC Assessment, multiple annual simulations were conducted using five GCMs, including the CESM2 (WACCM6) from the National Center for Atmospheric Research (Danabasoglu et al., 2020; Gettelman et al., 2019), the GFDL AM4.1 from the National Ocean and Atmospheric Administration (Dunne et al., 2020; Horowitz et al., 2020), the GISS E2.1 from NASA Goddard (Kelley et al., 2020), the MIROC-CHASER developed by the Atmosphere and Ocean Research Institute, University of Tokyo, the National Institute for Environmental Studies, the Japan Agency for Marine-Earth Science and Technology, and Nagoya University (Sekiya et al., 2018; Sudo et al., 2002; Watanabe et al., 2011), and the UKESM1 model developed by the UK Met Office and academic community (Archibald et al., 2020; Sellar et al., 2019).

In this work, we use ozone results from UNEP/CCAC simulations #1 and #2, the difference of which represents the annual tropospheric ozone response to an instantaneous and sustained 50% reduction in anthropogenic methane mixing ratios, while holding emissions of all other ozone precursors constant at 2015 levels. These and other analyses presented in the UNEP/CCAC Assessment show that ozone mixing ratios respond linearly to changes in methane mixing ratios of up ±556 ppbv, suggesting that the methane-ozone response ratios (i.e., O_3_ pptv/CH_4_ ppbv) derived from simulations #1 and #2 are also applicable to the range of methane perturbations tested here (~100 ppbv). Therefore, in this analysis, the methane-ozone responses derived from each of the five GCMs are formatted onto a common 0.5° × 0.5° grid and combined with annual global methane perturbations (Figure S1 in Supporting Information S1) to generate gridded timeseries of annual ozone changes in response to a 100 ppbv CH_4_ pulse in 2020 (Figure S1, right in Supporting Information S1). Figure S1 in Supporting Information S1 shows that the magnitude of the global ozone response varies across GCMs, however, Figure S2 in Supporting Information S1 also shows that the ozone response varies regionally, in part due to available ozone precursors. This motivates the need to use spatially explicit ozone-methane relationships as done here. Due to the atmospheric lifetime of methane and ozone, ozone concentrations across all regions are expected to return to their baseline values well before the end of the century (Figure S1, right in Supporting Information S1). To align with recent epidemiological studies, we use the MDA8 ozone exposure metric. We also average model results over the warmest sixth months in the Northern (April–September) and Southern (October–March) Hemisphere to capture peak ozone production months. Sections S1 and S2 in Supporting Information S1 provide further details on the calculation of the methane pulse and resulting maps of absolute summertime MDA8 O_3_ responses.

### 2.2. Population and Respiratory Mortality Characteristics

To estimate projections of total population and background respiratory mortality, our analysis draws on the Resources for the Future Socioeconomic Projections (RFF-SPs) data set. These data represent 1000 individual probabilistic projections for country-level population (Figure 1(3)) (Rennert et al., 2022b) and background all-cause mortality (Raftery & Ševčíková, 2023) (Figure 1(4)) from 2020 through 2300, stratified by age and sex. As described below, global estimated ozone-attributable respiratory-related mortality from a 2020 methane pulse is near negligible by the end of the century, such that we only rely on population and mortality data through the year 2100.

In this analysis, we focus on respiratory-related health endpoints as current epidemiological and toxicological research provides the strongest evidence for respiratory (vs. cardiovascular or other) health effects resulting from long-term exposure to ozone (U.S. EPA, 2020). Baseline mortality estimates in the RFF-SP data are not differentiated by cause of death. Therefore, to capture background respiratory-related deaths (Figure 1(5)) we scale RFF-SP country-level all-cause mortality projections using data from the International Futures Project (IFP) (International Futures (IFs) modeling system). The IFP includes projected country and age-specific estimates for both respiratory and all-cause deaths from 2000 through 2100. We take the ratio of these two as representative of the mortality fraction—by country, age, and year—projected to occur due to respiratory causes through the end of the century. We then multiply age- and country-specific all-cause mortality projections from RFF by the calculated respiratory-to-all-cause ratio projection from IFP data to derive the subset of deaths in each of the 1000 RFF-SP projections resulting from respiratory causes. Figure S3 in Supporting Information S1 shows the mean, 95th, and 99th percentile of the global population and derived global respiratory mortality rates from 2020 to 2100, with further calculation details in Section S3 in Supporting Information S1.

Individual projections of country-level population and derived respiratory-related mortality are then aggregated across sex and averaged across all 1000 trials for input into BenMAP. Annual country-level population data is additionally downscaled to a 0.5° × 0.5° global grid using population “cross-walks,” which represent the percentage of a given country’s population in each grid cell. We generate population cross-walks using the 2020 Gridded Population of the World (GPW) (Center for International Earth Science Information Network - CIESIN - Columbia University, 2018) at the 0.008° × 0.008° and 0.5° × 0.5° resolution. In contrast, mortality rates are not downscaled from country-level. Instead, BenMAP assigns a single mortality rate to all grid cells within each country, and calculates a population weighted average mortality rate for grid cells that intersect multiple countries.

### 2.3. Global BenMAP and Methane-Ozone Mortality

We use a new cloud-based version of U.S. EPA’s BenMAP to estimate global ozone-attributable respiratory-related mortality associated with a 2020 pulse of methane emissions. BenMAP was initially designed to estimate the incidence and value of health effects resulting from changes in air pollution in the United States. In addition to direct emission-air quality-health impacts, BenMAP has also been applied to climate-driven effects on air pollution and health within the U.S., such as the air quality health impacts associated with climate-driven changes in wildfire emissions (Neumann et al., 2021), southwest dust (Achakulwisut et al., 2019), pollen (Anenberg et al., 2017), heat (Morefield et al., 2018), and ozone and fine particulate matter (Fann et al., 2021) (though such climate-health related health impacts are not included in this study). More recently, the BenMAP tool was re-developed as a web application, in part to facilitate analyses with broad geographic scopes and finely resolved data inputs (Section S4 in Supporting Information S1). This analysis leverages these recent updates and represents the first study to estimate global air pollution health impacts using a global cloud-based version of this tool.

In this analysis, we use a log-linear health impact function within the global BenMAP framework to relate summertime MDA8 ozone exposure levels to the logarithm of respiratory deaths:

(1) $$y_{\text{ct}}={\text{Incidence }}_{\text{ct}}\times {\text{Population }}_{\text{ct}}\times \left(1-e^{-\beta {\Delta \text{O}}_{3}}\right)$$

where *y*_*ct*_ is the estimated change in annual respiratory-related deaths in 0.5° × 0.5° grid cell (*c*) and year (*t*). In Equation 1, *β* is the risk coefficient associated with ozone exposure and ΔO_3_ is the change in summertime MDA8 ozone mixing ratio. Lastly, Incidence_ct_ and Population_ct_ in Equation 1 represent gridded annual estimates of the baseline background respiratory mortality rates and total population counts, respectively, as described in Section 2.2., which are aggregated within BenMAP across all ages 0–99 years.

In this analysis, we applied a chronic obstructive pulmonary disorder (COPD) relative risk coefficient of 1.06 per 10 ppb ozone exposure (95% CI: 1.03, 1.10), as estimated by the Global Burden of Disease (GBD, 2019 Risk Factor Collaborators, 2020) (Figure 1(6)). This coefficient was derived from a meta-regression of five recent cohort studies in Canada, the United Kingdom, and the United States. Consistent with Malashock et al. (2022), we applied this COPD coefficient to all respiratory mortality in all countries. Epidemiological research suggests respiratory mortality from long-term ozone exposure is not limited to COPD. This body of literature includes Turner et al. (2016), one of the largest cohort studies used in the meta-regression described above.

BenMAP is then run with two ozone air quality surfaces for each year—baseline and methane-perturbed summertime MDAO_3_—the difference of which represents the change in mortality attributable to ozone produced from a 2020 methane emissions pulse (Figure S1 in Supporting Information S1). Maps of the resulting ΔMDA8 O_3_ mixing ratios and attributable deaths are then aggregated to the country level for the remainder of the analysis. Due to current computational limits in the new BenMAP webtool, simulations using ozone surfaces from each GCM are run every 5 years from 2020 to 2040 and every 10 years from 2040 through the end of the century. Country-level mortality results are then interpolated between these years to derive the complete timeseries of attributable respiratory mortality counts (Figure S4 in Supporting Information S1).

### 2.4. Monetization of Methane-Ozone Mortality

This analysis uses VSL estimates to monetize the costs associated with chronic respiratory-related deaths each year attributable to changes in ozone from a 2020 methane emissions pulse. In this context, VSL refers to an individual’s willingness to pay for a small reduction in the risk of their own premature death within each future year, calculated as the population average for each country. This analysis does not include non-mortality-related costs, such as direct spending on health care or any environmental effects on labor productivity. Annual country-level damages associated with methane-ozone mortality estimates are calculated using the country- and year-specific VSL estimates, shown in Equation 2, which represents the cost an individual would be willing to pay to reduce the risk of mortality.

(2) $${\text{VSL}}_{c,t}={\text{VSL}}_{\text{US},2020}\times {\left(\frac{{\text{Income }}_{c,t}}{{\text{Income }}_{\text{US},2020}}\right)}^{\varepsilon }$$

Since present and future estimates of VSL are not available for each country and region, we calculate the VSL for each country (*c*) and year (*t*), by referencing to the EPA 1990 VSL for the U.S. (U.S. EPA, 2010) (adjusted for income growth and inflation to $10.05 million in 2020 dollars (U.S. EPA, 2022)), and scaling relative to U.S. income in 2020. We also set the income elasticity (*ε*) to 1, following Hammitt and Robinson (2011) and Rennert et al. (2022a), such that the estimated VSL is proportional to income in each country. Due to limited availability of socioeconomic projections, we approximate future changes in income as GDP per capita, consistent with previous similar studies, using projections of country-specific GDP and population data from the RFF-SP data set (Rennert et al., 2021, 2022b). Our central estimate presented in Section 3.2 uses the average population, background mortality, and GDP across all 10,000 projections. Annual monetized damages each year are then calculated as annual mortality counts for each country, multiplied by the country-level annual VSL estimates. We test the sensitivity to the range of socioeconomic conditions in Section 3.

The full stream of monetized annual impacts from chronic respiratory mortality from methane-ozone are then discounted back to the year of emissions (2020) and integrated to calculate the Net Present Value (NPV). Discounting converts future impacts into present dollar equivalents, accounting for the fact that each dollar in the future is typically valued less than in the present. NPV calculations can be highly sensitive to discount rate and approach used, though less so for shorter lived gases like methane than for long-lived gases like CO_2_. Therefore, we test the sensitivity to both a constant and Ramsey discounting approach. While the former applies a constant discount rate over time (effectively assuming *η* = 0), the Ramsey approach in Equation 3 allows the discount rate to scale over time with future economic growth, such that impacts are more highly valued in futures with low economic growth. The time-varying and state-specific Ramsey discount rate follows Equation 3

(3) $$\text{Ramsey discounting }{\text{factor}}_{t}=\rho +\eta g_{t}$$

where (*g*_*t*_) is per capita economic consumption growth in each country from the year of the emissions pulse to year *t*, *ρ* is the pure rate of time preference, and *η* is the elasticity of the marginal value of consumption with change in *g*_*t*_. We calculate the stochastic Ramsey discount factor (Section S5 in Supporting Information S1) and apply the resulting time-varying rate in Equation 4, such that the NPV in each country is

(4) $$NPV=\sum_{t=2020}^{t=2100}\frac{\text{Annual }{\text{damages}}_{t}}{\prod_{x=2020}^{x=t}\left(1+\text{Ramsey discount }{\text{factor}}_{x}\right)}$$

This approach has been used in recent NPV analyses of climate health related damages (Hartin et al., 2023) and is generally consistent with the social cost of carbon framework, recently applied in Rennert et al. (2022a). However, for consistency with country-specific VSL estimates, this analysis uses discount factors based on country-level consumption growth rather than the world average, which results in a more conservative NPV estimate (Section S5 in Supporting Information S1). Our central estimate focuses on results discounted using time-varying Ramsey discount rates, calibrated to a near-term discount rate of 2.0%. Additional details are described in Section S5 in Supporting Information S1. All results in this analysis are presented in units of 2020 U.S. dollars, converted from 2011 values (RFF-SP dollar units) using Annual GDP Implicit Price Deflators (U.S. Bureau of Economic Analysis, 2023).

### 2.5. Reduced Form Model

To further assess the sensitivity of the monetized damages to alternative socioeconomic projections and emission scenarios, we supplement the BenMAP analysis with a custom reduced form tool. The reduced form model is an R-based tool that adjusts the BenMAP generated attributable mortality counts to produce new estimates of annual country-level methane-ozone attributable respiratory-related deaths from a pulse of methane emissions, following Equation 5:

(5) $${\text{Mortality}}_{c,t,p}={\text{ Mortality}}_{c,t,b}\times \left(\frac{{\text{Incidence}}_{c,t,p}}{{\text{Incidence}}_{c,t,b}}\right)\times \left(\frac{{\text{Population}}_{c,t,p}}{{\text{Population}}_{c,t,b}}\right)\times \left(\frac{{\text{O}}_{3}\;{\text{Response}}_{c,t,p}\times {\text{CH}}_{4}\;{\text{Pulse}}_{t,p}}{{\text{O}}_{3}\;{\text{Response}}_{c,b}\times {\text{CH}}_{4}\;{\text{Pulse}}_{t,b}}\right)$$

where the updated mortality estimates for each country (*c*) and year (*t*) and for each new projected scenario (*p*) are equal to the original annual mortality estimates from BenMAP (*b*), scaled by the ratio of the background respiratory mortality incidence, total population, and summertime ΔMDA8 O_3_ in the new projected scenario relative to those in the original BenMAP simulations. In Equation 5, the ratio of summertime MDA8 O_3_ levels is calculated as the average O_3_ response to methane (O_3_ pptv/CH_4_ ppbv) across each country and year, multiplied by annual ΔCH_4_ concentrations from an emissions pulse in a given year. The O_3_ response in the original BenMAP simulations are assumed constant over time and the annual perturbed CH_4_ concentrations in any new scenario are calculated using the pulse size and atmospheric lifetime of CH_4_, as discussed in Section 2.1 (Figure S1 in Supporting Information S1).

While the formulation in Equation 5 assumes linear relationships at the country level between changes in perturbed ozone, population characteristics, and attributable deaths, the efficiency of tropospheric O_3_ production from atmospheric methane (i.e., O_3_ response) is sensitive to changes in O_3_ precursors, such as nitrogen oxides (NO_x_ = NO + NO_2_). Therefore, the logarithmic relationship in Equation 6 can be used to relate changes in NO_x_ emissions to changes in the O_3_-methane response in each country. We leverage the relationships derived as part of the UNEP/CCAC Global Methane Assessment, from two additional sets of simulations that assessed the change in O_3_ response with methane at varying NO_x_ emission levels (UNEP/CCAC, 2021).

(6) $$\frac{\Delta {\text{MDA}}_{8}{\text{O}}_{3}\left(\text{pptv}\right)}{{\text{CH}}_{4}\left(\text{ppbv}\right)}=\frac{1000\left(\text{slope}\times \text{ln}\left({\text{NO}}_{\text{x}}\right)+\text{intercept }\right)}{556\text{ ppbv}}$$

The resulting annual country level mortality estimates from the reduced form tool (under any custom scenario) can be monetized, discounted, and aggregated using the methods described in Section 2.4. Sensitivities of annual monetized and discounted NPVs to changes in socioeconomic and NO_x_ emission projections, as predicted by the reduced form tool, are presented in Section 3.

## 3. Results and Discussion

### 3.1. Physical Impacts

**Figure 2.**
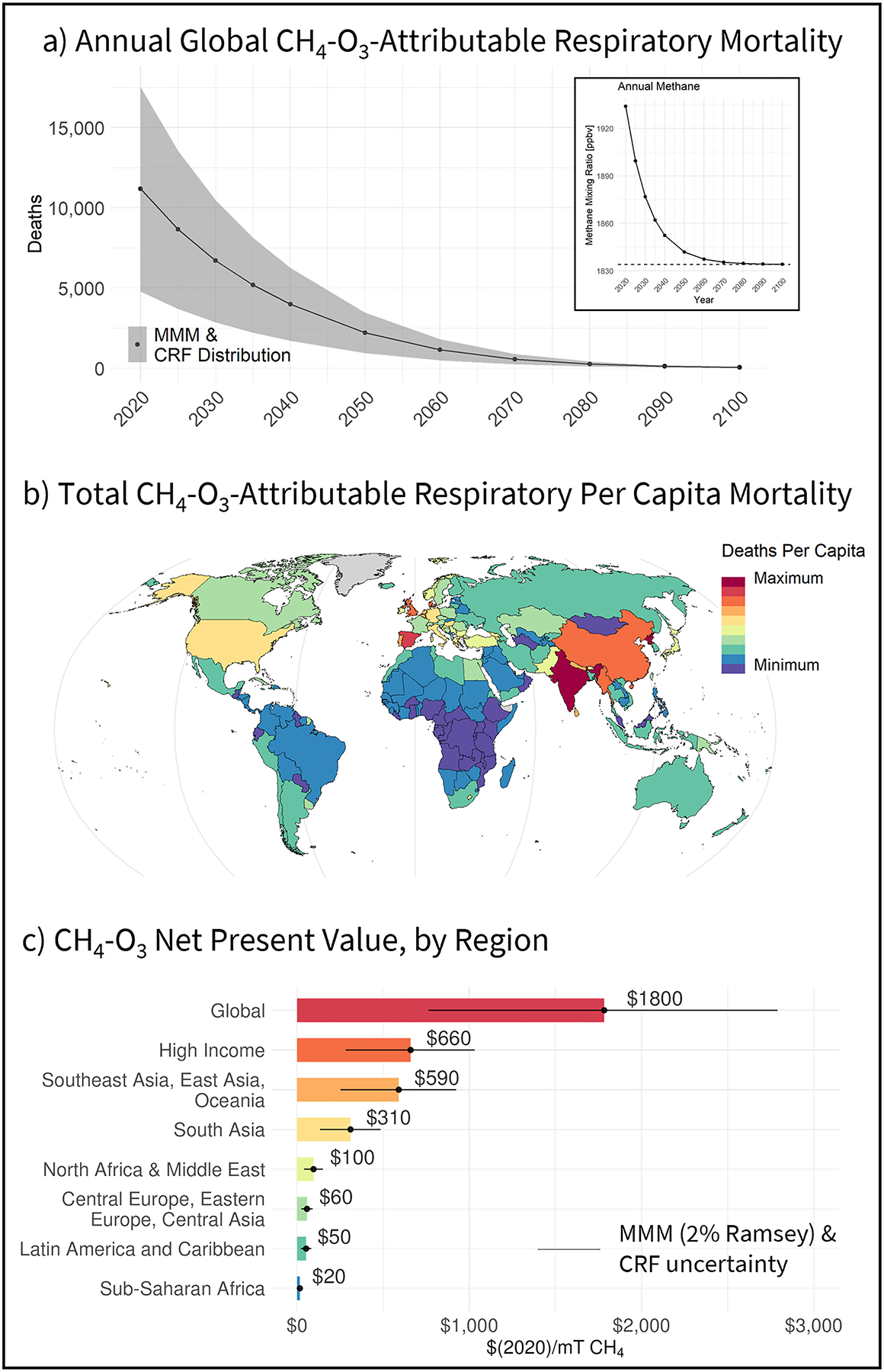
Physical and economic impacts of ozone produced from a 275 MMT pulse of methane emissions in the year 2020. (a) Timeseries of annual global respiratory-related deaths attributable to O_3_ exposure (with CRF uncertainty) and timeseries of methane mixing ratio (insert), (b) respiratory-related deaths per capita attributable to ozone in 2020, by country, (c) net present value of methane-ozone attributable respiratory related deaths (with CRF uncertainty), globally and by GBD Super Region.

Globally by the end of the century, an estimated total of 210,000 (95% Confidence Interval: 90,000–330,000) respiratory related deaths would be attributable to tropospheric ozone produced from a 275 million metric ton (MMT) pulse of methane emissions in 2020. Figure 2a illustrates that, in the absence of cessation lags, annual mortality counts peak in the same year as the initial emissions pulse, which also coincides with the timing of the largest perturbations in methane and ozone concentrations (Figure S1 in Supporting Information S1). Annual physical impacts are calculated directly by the global BenMAP webtool, using average population and respiratory mortality rate projections as described in Section 2 and the ΔMDA8 summertime O_3_ mixing ratios per change in methane mixing ratio from the mean of the five GCMs (MMM). Uncertainty in the GBD ozone concentration response function (CRF) underlying BenMAP (β 95% CI: 1.03–1.10 per 10 ppbv O_3_) is shown by the 95th percent confidence interval in Figure 2a. Annual estimates are also sensitive to differences in the methane-ozone response in each GCM (Figures S4 and S5 in Supporting Information S1) and range from a total of 140,000 deaths through the end of the century predicted by the MIROC model, up to 320,000 total attributable deaths predicted by HadGEM (95% CI: −43% to +56% for both), given average population characteristics. A discussion of these and additional uncertainties associated with socioeconomic projections, precursor emissions, and valuation are discussed in Section 3.3.

Figure 2b additionally illustrates that CH_4_-O_3_ attributable respiratory-related deaths are not distributed evenly across countries and regions. As BenMAP applies the same ozone concentration response function to all regions, heterogeneity in mortality counts across countries is driven by a combination of differences in country-level population, background respiratory mortality rates (Equation 1), as well as differences in the modeled ozone response to methane change (Figure S2 in Supporting Information S1). While absolute population is the main driver of these differences (Figure S6a in Supporting Information S1), by normalizing mortality counts per capita in Figure 2b, the remaining spatial differences illustrate that additional differences in regional background respiratory mortality rates and ozone response to methane are also important factors. For example, while highly populated countries in the South Asia “GBD Super Region” (Table S1 in Supporting Information S1) are estimated to collectively have the largest total attributable mortality counts (40% of global total), panels b–c in Figure S6 in Supporting Information S1 also show that countries in this region have higher background mortality rates and a more sensitive ozone response to methane (~4.6 pptv O_3_/ppbv CH_4_) relative to the population-weighted global modeled average (4.1 pptv O_3_/ppbv CH_4_) (e.g., Figure S2 in Supporting Information S1). Likewise, relatively lower deaths per capita in central Africa are in part due to relatively lower respiratory mortality rates and less efficient methane-ozone production (Figure S6 in Supporting Information S1). While West et al. (2006) previously showed all-cause per capita methane-ozone impacts were greatest in countries within the Africa region, that study similarly found that per capita cardiovascular and respiratory-related mortality impacts were relatively greater throughout Europe. Despite differences in magnitude (discussed below) these patterns are generally consistent with the relative spatial patterns in the respiratory-related mortality estimates in this study. The UNEP/CCAC Global Methane Assessment likewise reported similar spatial patterns in cardiovascular and respiratory-related mortality estimates to those shown here, other than for Sudan (UNEP/CCAC, 2021).

Lastly, due to the linear relationship between changes in atmospheric methane and ozone, we scale total integrated deaths from our original pulse down to 760 (95% CI: 330–1200) total deaths per MMT of CH_4_. The deaths/MMT results from this work are slightly larger, but comparable to previous similar studies. For example, the UNEP/CCAC Global Methane Assessment estimated 740 (95% CI: 460–990) respiratory-related attributable deaths per MMT CH_4_, as well as an additional 690 (95% CI: 210–1120) attributable deaths from cardiovascular diseases (UNEP/CCAC, 2021). Though these values are derived from the same GCM simulations used in this work, respiratory estimates slightly vary from those presented in this study due to differences in the β, minimum exposure limit (Section S4 in Supporting Information S1), and assumptions of constant 2015 populations and mortality rates relative to dynamic population projections used here. Additional sensitivities to non-respiratory health endpoints are discussed in Section 3.3. In contrast, Sarofim et al. (2017) estimated 239–591 deaths/MMT, which is smaller than estimates here in part due to the spatially homogenous methane perturbation assumption used in that study. Assuming a homogeneous, globally averaged methane-ozone response across all grid cells in our study also results in lower mortality estimates, which fall within the Sarofim et al. (2017) range. Lastly, all-cause mortality estimates from methane-ozone derived from West et al. (2006) are close to 300 deaths/MMT, which may be lower than our estimates due to differences in modeling approach, a lower average simulated methane ozone response and *β*, and different assumptions in projected population and mortality characteristics. Results are sensitive to these parameters, and we discuss the sensitivity to each below.

### 3.2. Economic Damages

As described in Section 2, annual streams of attributable deaths in each country are monetized, discounted back to present day values, and integrated to derive a NPV of the total economic damages associated with ozone-attributable respiratory-related deaths per mT of methane emissions. Due to the linear relationship between atmospheric methane and ozone changes, we linearly scale the total integrated discounted damages from our original 275 MMT (or 100 ppbv) pulse down to units of dollars per metric ton (mT) of CH_4_.

Globally, the central NPV derived from the MMM and using a 2% Ramsey discount rate is $1800/mT CH_4_ (95% CI: $760–$2800/mT CH_4_). The 95% confidence interval is associated with the upper and lower bounds of the ozone exposure response function in the global BenMAP webtool. Mean NPV results are most sensitive to these BenMAP uncertainties. These and additional sensitivities are discussed in the following section. Similar to the regional trends in physical impacts, the total economic damages related to methane-ozone mortality are not evenly distributed across world regions (Figure 2c). As anticipated, large NPV values are estimated across regions that also have large attributable mortality counts, however, net present damages are estimated to be largest in the “High Income” region ($660/mT CH_4_; 95% CI: $280–$1030/mT CH_4_), in part because of regional differences in projected income. These large values in the high-income region are driven by large NPV’s in the U.S., Japan, and throughout western Europe (Table S1 in Supporting Information S1). The region with the second highest aggregate NPV is the Southeast Asia, East Asia, and Oceania region ($590/mT CH_4_; 95% CI: $250–$920/mT CH_4_), driven by high values in China, followed by the South Asia ($310/mT CH_4_; 95% CI: $130–490/mT CH_4_) and North Africa and Middle East regions ($100/mT CH_4_; 95% CI: $40–$150/mT CH_4_). NPV’s for the top 20 countries are shown in Table S1 in Supporting Information S1.

Given sensitivities to differences in assumptions regarding discount rates, concentration response functions for mortality, VSL estimates, and other factors, results from previous studies can be challenging to compare with more recent numbers, particularly for older studies such as West et al. (2006). Even for newer studies, there are many differences in assumptions that drive the differences between estimated valuations. For example, UNEP/CCAC (2021) estimated a value of (2020) $2580/mT CH_4_ including cardiovascular deaths with a value of $1335/mT CH_4_ for respiratory deaths only, as in this study, similar to the value reported here. Their calculation used a constant discount rate of 3%, and didn’t include future increases in population, which may account for the slightly lower valuation. Sarofim et al. (2017) presented a range of (2020) $900–$2100/mT CH_4_, within the range of results here, despite projecting fewer deaths and using a higher discount rate. However, the elasticity of VSL estimates to GDP/capita used in Sarofim et al. (2017) was 0.4, which both Sarofim et al. (2017) and UNEP/CCAC (2021) have shown leads to a doubling of the damage estimate relative to an elasticity of 1 used here. When using a monetization and discounting approach consistent with the updated social cost of carbon framework, our monetized impacts of ozone per mT of CH_4_ are larger than the current SC-CH_4_ estimates of $1500/mT (3% CDR) used by the U.S. government (Interagency Working Group on Social Cost of Greenhouse Gases (IWG), 2021), as well as the recently updated estimates of $1600/mT CH_4_ (2% Ramsey) (EPA, 2022), both of which are only based on climate-related damages.

### 3.3. Uncertainties and Sensitivities

**Figure 3.**
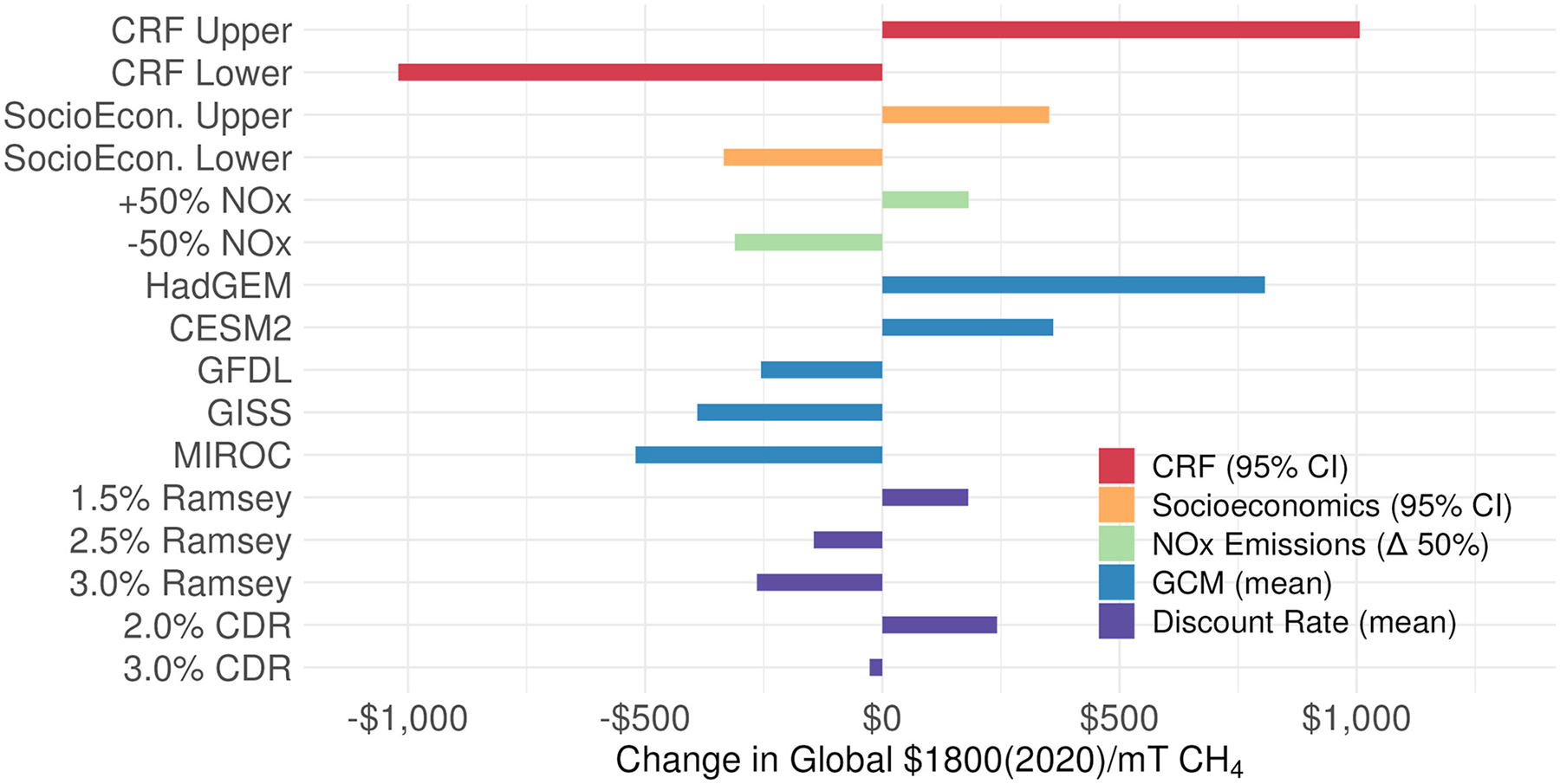
Sensitivity of the mean global NPV to uncertain analysis parameters. The top four bars represent the ranges associated with the 95% confidence interval of the BenMAP concentration response function (CRF) (red) and RFF-SP socioeconomic projections (orange). The remaining bars represent changes in the mean value associated with ±50% changes in NO_x_ emissions (green), differences across five GCMs (blue), and five discounting rates and approaches (Ramsey and constant discount rates) (purple). Socioeconomic and NO_x_ sensitivity results were derived from runs with the reduced form tool, while remaining sensitivities were derived from the central BenMAP run. Note, these parameters are only a partial accounting of all NPV uncertainties, as discussed in the main text.

Consistent with previous approaches to estimating the social cost of greenhouse gases, there are many sources of uncertainty in estimating the physical and economic impacts from ozone produced from a ton of methane emissions. Major sources of uncertainty include but are not limited to: climate model representation of atmospheric conditions that drive ozone production from methane, the sensitivity of ozone production chemistry to precursor emissions, projections of country-level GDP, population counts and total all-cause and cause-specific mortality rates through the end of the century, changes in the respiratory-related health risk associated with changes ozone exposure, as well as the discount approach and rate used to monetize the full stream of annual damages. Figure 3 summarizes the sensitivity of the global NPV to these major sources of uncertainty, which are discussed in order of decreasing sensitivity below.

#### 3.3.1. Concentration Response Function

The global NPV from respiratory-related deaths attributable to methane-produced ozone is sensitive to uncertainties in the ozone concentration response function (β) implemented in BenMAP. As shown in Figure 2a, the 95% confidence interval of β values from the GBD (1.03–1.10/10 ppbv O_3_ (GBD, 2019 Risk Factor Collaborators, 2020)) results in a range of total integrated mortality counts of 90,000–330,000 (mean: 210,000 deaths), which corresponds a change in global NPV of −57% to +56% (or $760–$2800/mT CH_4_) (Figure 3). Additional related uncertainty not considered here also arises from the application of the COPD hazard ratio to respiratory mortality (as described in Section 2.3), provided the COPD ratio includes more diseases, but is the best available at the global scale.

#### 3.3.2. Socioeconomics

Due to the computational requirements to run the global BenMAP webtool for each simulation year, climate model air quality surface, and future population and mortality projection, we alternatively develop a computationally efficient reduced form tool that can facilitate SC-CH_4_ calculations and can be run with any of the 10,000 probabilistic socioeconomic projections from the RFF-SPs (Raftery & Ševčíková, 2023; Rennert et al., 2021). Additional runs for specific projections with the BenMAP tool show that the reduced form tool can reproduce BenMAP respiratory-related deaths to within 0.5% (Section S6 in Supporting Information S1). We run the tool for all 10,000 future scenarios here to test the sensitivity of the mean NPV to the range of future socioeconomic (total population, mortality rates, GDP) projections. Figure 3 shows that across all future RFF-SP scenarios of country-level socioeconomic data, the 95% confidence interval of the global NPV with a 2% Ramsey discount factor is −18% to +19% (or $1500–$2200/mT CH_4_). As an additional evaluation of the reduced form tool, the mean NPV resulting from all 10,000 individual trajectories is within 1.5% of the NPV derived from the mean BenMAP run, which used a single projection of population, mortality, and GDP, calculated as the average of all 10,000 RFF-SP scenarios.

#### 3.3.3. Ozone Production Chemistry (Global Climate Model and Precursor Emissions)

The atmospheric production of tropospheric ozone requires the presence of NO_x_, volatile organic compounds (VOC) or carbon monoxide (CO), and sunlight. The efficiency of this non-linear relationship depends on the relative abundance of precursors, as well as factors that affect photochemical rates (i.e., temperature, sunlight, surface reflectance, etc.), such that O_3_ production may become more or less sensitive to changes in background methane levels depending on these conditions. As described in the UNEP/CCAC Global Methane Assessment, global simulations of tropospheric ozone changes in response to methane reductions were run with five GCMs. As each model incorporates different parameterizations of the physical and chemical conditions driving tropospheric ozone production, each model predicts a different level of absolute ozone change in response to global methane reductions (Figure S1 in Supporting Information S1), as well as a different spatial pattern of this response (Figure S2 in Supporting Information S1).

In this work, maps of summertime MDA8 O_3_ resulting from a 2020 CH_4_ emissions pulse are calculated using 0.5° × 0.5° gridded O_3_/CH_4_ response relationships derived from UNEP/CCAC simulations (assuming a constant response relationship over time). Therefore, to test the sensitivity of the economic impacts to the choice of GCM, we run the BenMAP webtool with O_3_ maps calculated from the ozone response in each of the five GCMs. Our central value is from the multi-model mean (MMM). As shown in Figure 3, the five GCMs result in a spread of global NPV values (with 2% Ramsey discount factor) of −30% to +45% (or $1300–$2600/mT CH_4_) relative to the MMM.

In addition to GCM chemistry and parameterizations, the chemical response of O_3_ production to changes in background methane levels (e.g., pptv O_3_/ppbv CH_4_) is also sensitive to the relative abundance of NO_x_ and VOC + CO precursor emissions. As shown in the UNEP/CCAC Global Methane Assessment, methane emission changes will have a smaller impact on ΔMDA O_3_ as regional NO_x_ emissions are reduced and ozone photochemistry becomes more NO_x_-limited (i.e., VOC saturated). In contrast, methane will have a larger impact on ΔMDA8 O_3_ as NO_x_ emissions increase, and ozone photochemistry becomes more VOC-limited. Despite the complex non-linear nature of this chemistry, an additional set of UNEP/CCAC simulations using varying NO_x_ emissions showed that the ozone response to changes in methane generally follows a log-linear relationship with changes in absolute NO_x_ emissions (Equation 6), but that the slope and intercept of this relationship varies by country. The ozone-methane sensitivity was also found to be much weaker for changes in other VOC emissions, such that no relationship was derived. Previous simulations by West et al. (2006) also found a low sensitivity of the ozone-methane response to changes in either NO_x_ and VOC precursor emissions. Here we test the sensitivity to changes in NO_x_ emissions by parameterizing the methane-ozone response relationship in the reduced form tool using the NOx-O_3_/CH_4_ relationship for each country, derived from the UNEP/CCAC simulation results (Equation 6). Simulating a 50% change in NO_x_ emissions in each country relative to original model levels (from UNEP/CCAC simulations) results in a NPV change (MMM, 2% Ramsey discount factor) of −17% to +10% (or $1500–$2000/mT CH_4_). Additional sensitivity to changes in NO_x_ emissions over time were not tested here but could be implemented in the reduced form tool (Section S6 in Supporting Information S1) and are expected to have a relatively smaller impact on discounted future damages. These combined results suggest that damages associated with mortality attributable to methane-produced ozone are more highly sensitive to choice in GCM rather than the impacts of NO_x_ emissions on photochemical methane-ozone production efficiency.

Additional uncertainties include the sensitivity to model resolution, as well as the change in NO_x_/VOC sensitivity in a region over time, and the contribution of methane to localized ozone production (e.g., <1 km scale). Therefore, while this analysis is generally consistent with the global SC-GHG framework, the approach used here is less relevant for resolving highly localized air quality benefits.

#### 3.3.4. Monetization

Consistent with recent analyses of the social cost of greenhouse gases (Rennert et al., 2022a), the NPV’s in this analysis are also sensitive to parameters used to monetize the economic damages associated with changes in mortality. These include the base VSL, estimates of future income growth, income elasticity, and discounting approach. As discussed in Section 2.4, parameters used for the central NPV in this analysis are chosen to align with the current social cost framework (Rennert et al., 2022a), such that the base VSL = $10.05 million, *ε* = 1, and future income is approximated as GDP per capita. However, as monetization of mortality risk is an active area of research, it remains important to consider sensitivities to these parameters. For example, NPV estimates are directly proportional to changes in base VSL, as shown in Equation 2, such that ±20% changes in base VSL would result in ±20% changes in the NPV. In addition, while the current SC-GHG framework uses an income elasticity (*ε* = 1) based on the central tendency in recent literature (e.g., Hammitt & Robinson, 2011; Rennert et al., 2022a), the research on elasticity is unsettled (e.g., Masterman & Viscusi, 2018, 2020). Testing a range of previously proposed values of 0.4 (Sarofim et al., 2017; UNEP/CCAC, 2021) to 1.5 (Robinson et al., 2019) across all countries results in a change in the global mean NPV of −25% (*ε* = 1.5) to +75% (*ε* = 0.4). Lastly, we follow the recent approach of Rennert et al. (2022) and also present the sensitivity of the mean NPV to differences in discounting approach and rate. As shown in Figure 3 (and Figure S7 in Supporting Information S1), the central global mean NPV is modestly sensitive to the discount approach and factor used (constant discount factor vs. time-varying Ramsey approach). The central mean value in this analysis uses the 2.0% Ramsey discount factor approach but ranges from $1500/mT CH_4_ with a 3.0% Ramsey discount factor up to $2000/mT CH_4_ with a 2% constant discount factor. Discount factors are calculated at the country-level. Aggregated regional NPV’s across all discount factors tested here are shown in Figure S7 in Supporting Information S1.

#### 3.3.5. Additional Uncertainties and Limitations

Additional uncertainties that are not included in Figure 3 include the possible delay between initial ozone exposure and the year when death is estimated to occur (cessation lags) and the minimum exposure level under which there is no additional risk from ozone exposure (TMREL). The global total mortality counts from the MMM are only minorly sensitive to the TMREL (−3%, Section S4 in Supporting Information S1), and implementation of cessation lags only reduce the global NPV by 2.5% (Section S5 in Supporting Information S1) relative to the MMM. Additional uncertainties also include mortality that might occur due to exposure in the winter months or the consideration of damages from additional health endpoints, such as cardiovascular-related mortality, or morbidity outcomes such as increased hospitalizations or asthma-related emergency department visits. While this current study is designed to align with recent U.S. EPA causality determinations for respiratory and cardiovascular-related mortality from long-term exposure (U.S. EPA, 2020) (Section S4 in Supporting Information S1), results presented in the UNEP/CCAC Assessment also suggest that additional non-respiratory health endpoints (particularly mortality impacts) may contribute to additional physical and monetized impacts not captured here. However, any additional impacts will be highly dependent on future projections of country- and disease-specific baseline mortality rates and the availability of baseline data for morbidity outcomes (UNEP/CCAC, 2021). There are also uncertainties associated with the epidemiologic studies underlying the respiratory-related estimates of ozone exposure risk used here. Some of these include using a pooled hazard ratio from a limited number of studies in developed countries and applying that to the countries in the developing world, as well as using historical associations between exposure and adverse effects to quantify these risks in the distant future. These and additional sensitivities are not tested here but could, in part, be explored using a range of input parameters in the reduced form tool (Section S6 in Supporting Information S1).

One additional potential benefit of the reduced form model is the ability to assess methane perturbation results from external climate models such as FaIR (Leach et al., 2021). In this paper, a constant methane lifetime of 11.8 years was used, but future methane lifetime is a function of future emissions of VOCs, NO_x_, and methane itself, as well as of changes in global temperature and other factors. A note of caution, however, is that the factors impacting the methane lifetime would also be expected to change the ozone production relationship, and besides the NO_x_ sensitivity analysis discussed above, the reduced form model doesn’t have any ability to account for the effects of these other changes.

## 4. Conclusions

This analysis combines the SC-CH_4_-relevant best practices of earlier papers (including the use of future population characteristics as in Sarofim et al. (2017), heterogenous ozone response as in UNEP/CCAC (2021), and socioeconomic and population projections from Rennert et al. (2021)), in order to estimate an SC-CH_4_-consistent set of damages resulting from ozone produced from CH_4_ emissions. The global NPV magnitude ($1800/mT CH_4_) is comparable in size to the most recent climate-based SC-CH_4_ estimates. The NPV is sensitive to uncertainties in the health impacts of ozone exposure, parameterized ozone production chemistry in GCMs, and assumptions in future socioeconomic conditions. The additional development of a reduced form model, based on detailed underlying climate-chemistry and health impact models, allows this work to be coupled to alternative assumptions about future populations, mortality rates, precursor emissions, pulse year, and monetization assumptions (such as the base VSL, the elasticity of VSL estimates with income, and the discount rate). This could enable integration with SC-CH_4_ estimation frameworks such as the GIVE model (Rennert et al., 2022a). These advances are potentially an important step to including these effects in future cost-benefit analyses.

## Supplementary Material

SI

## Data Availability

The Global BenMAP model instance (Version 1) used in this analysis is publicly available on Zenodo (McDuffie et al., 2023a). A repository (Version 1.0) that contains the reduced form model source code, all inputs (including UNEP/CCAC ozone output), results, and analysis and figure scripts used in this manuscript is licensed under MIT and Creative Commons and published on GitHub (McDuffie et al., 2023b).

## Key Points:

- Increases in mortality attributable to ozone produced from methane are not currently considered in the government’s social cost of methane
- Ozone from a 2020 methane emissions pulse results in 760 deaths per million metric ton and a net present value of $1800 per metric ton
- A reduced form tool is developed to assess uncertainties and facilitate additional social cost of methane calculations
