# Supplementary material for: The Social Cost of Ozone-Related Mortality Impacts From Methane Emissions
Source: Earths Future. Author manuscript; Available in PMC 2023 Nov 7. (PMC10631284; doi:10.1029/2023ef003853)
Supplement: SI [file NIHMS1940316-supplement-SI.pdf]

AGU's Earth's Future

Supporting Information for:

The Social Cost of Ozone-Related Mortality Impacts from Methane Emissions

Erin E. McDuffie[1], Marcus C. Sarofim[1], William Raich[2], Melanie Jackson[2], Henry Roman[2], Karl Seltzer[3], Barron Henderson[3], Drew T. Shindell[4], Mei Collins[2], Jim Anderton[2], Sarah Barr[1], Neal Fann[5]

[1]*Office of Atmospheric Protection, Climate Change Division, U.S. Environmental Protection Agency, Washington, DC, USA*
[2]*Industrial Economics, Incorporated, Cambridge, MA, USA*
[3]*Office of Air Quality Planning and Standards, Air Quality Assessment Division, U.S. Environmental Protection Agency, Research Triangle Park, NC, USA*
[4]*Nicholas School of the Environment, Duke University, Durham, NC, USA*
[5]*Office of Air Quality Planning and Standards, Health and Environmental Impacts Division, U.S. Environmental Protection Agency, Research Triangle Park, NC, USA*

Contents of this File

Text S1 to S6
Figures S1 to S7
Tables S1 to S2
References

Introduction

This supporting information provides the supplemental figures, tables, and text sections that are referenced in the main text, including detailed information on the calculation of atmospheric methane and ozone concentrations, population characteristics, BenMAP mortality estimates, monetization approach, and reduced-form tool.

Text S1. Atmospheric Methane Changes

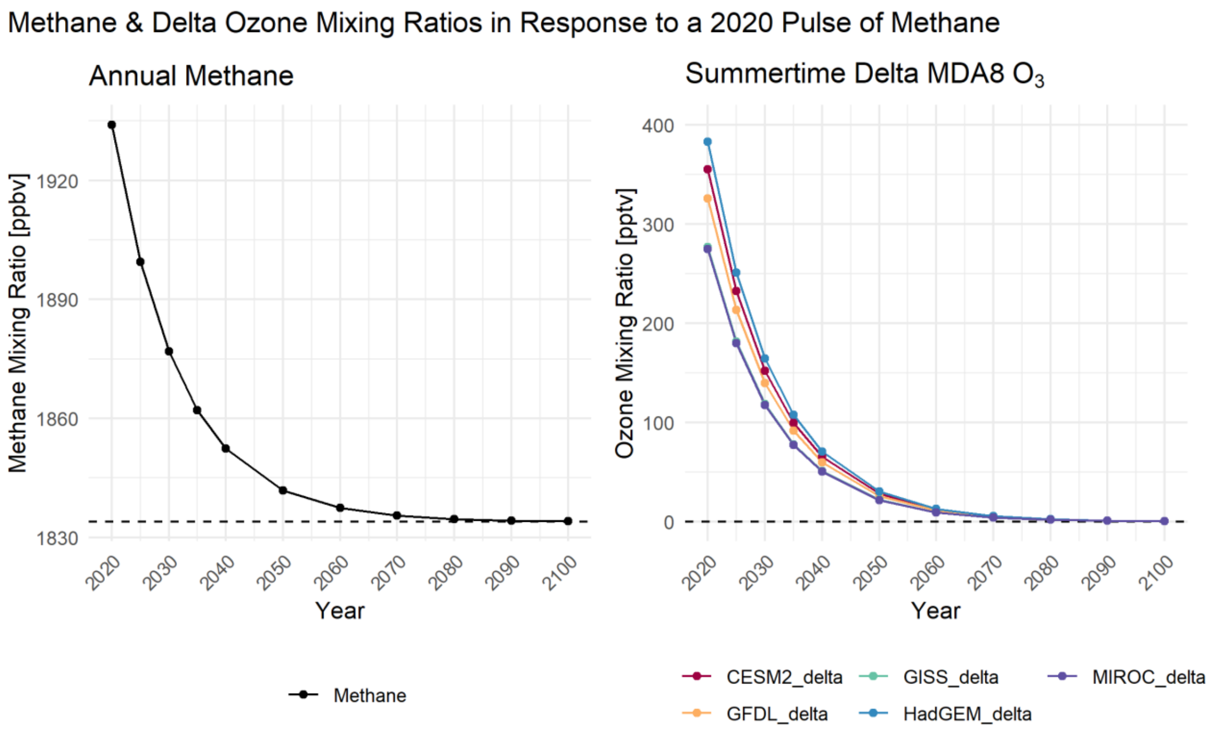

Figure S1. Timeseries of perturbed annual average atmospheric methane (left) and Δ summertime maximum daily 8-hour average (MDA8) ozone mixing ratios (right), in response to a 275 MMT pulse of methane in the year 2020. ΔMDA8 ozone results are shown for each model. The baseline CH_4 mixing ratio (1834 ppbv) is shown by the left dashed line.

The first step in the analysis workflow shown in Figure 1 is to estimate the perturbations in atmospheric methane concentrations through the end of the century, associated with a pulse of methane emissions in the year 2020. We chose a pulse size of 275 million metric tons (MMT) of CH_4, which corresponds to a mixing ratio of ~100 ppbv following the 2.75 Tg/ppbv CH_4 conversation factor, from *Prather et al.* [2012], used in the 5th Assessment Report of the IPCC.

Reproduced from *Prather et al.* [2012] Supplemental Text S1:

$$2.75\ \frac{\mathrm{Tg\ CH_4}}{\mathrm{ppbv}} = 0.1764\ \frac{\mathrm{Tmoles\ air}}{\mathrm{ppbv}} * 16\ \frac{\mathrm{Tg\ CH_4}}{\mathrm{Tmoles\ CH_4}} * 0.973\ \frac{\mathrm{Tmoles\ CH_4}}{\mathrm{Tmoles\ air}} \qquad \text{(Eq. S1)}$$

As methane will exponentially decay in the atmosphere (unless resupplied), we use the perturbation lifetime of methane ($\tau = 11.8$ years) from IPCC AR6 [*Szopa et al.*, 2021] in Eq. S2 to derive the timeseries of perturbed methane mixing ratios shown in Figure S1. In Eq. S2, the initial pulse of methane

is 100 ppbv and the baseline is 1834 ppbv, from the UNEP/CCAC Global Methane Assessment simulations [UNEP/CCAC, 2021].

$$\text{Perturbed } [\text{CH}_4]_t = \text{ Pulse } [\text{CH}_4]_{t=0} e^{-\Delta t/\tau} + \text{Baseline } [\text{CH}_4] \quad \text{(Eq. S2)}$$

Text S2. Calculated Tropospheric Ozone Changes

As described in the main text, the timeseries of perturbed methane mixing ratios associated with a pulse of emissions in 2020 is combined with methane-ozone response maps (i.e., O_3 pptv/CH_4 ppbv) from the UNEP/CCAC Global Methane Assessment to calculate spatially explicit maps of ozone concentrations over time, in response to the initial 2020 CH_4 pulse. As discussed in the UNEP/CCAC Global Methane Assessment Report, the ozone response per ppbv of methane change is linear across the range of methane changes analyzed in the report (± 556 ppbv), which is larger than the 100 ppbv pulse size tested here. While linear, the magnitude of the ozone response to methane does vary regionally, as discussed below. We use the UNEP/CCAC O_3 response maps from each of the 5 GCMs used in the UNEP/CCAC Assessment, as well as resulting ozone concentrations calculated from the mean across all models (MMM). The gridded methane-ozone response relationships used to calculate these maps are derived from the changes in O_3 and CH_4 between simulations #1 and #2 from the UNEP/CCAC Assessment.

The calculated (unweighted) ozone responses to a 100 ppbv methane pulse for each model in the year 2020 are provided in Figure S2. The global population-weighted responses for each model are as follows (in pptv O_3 / 100 ppbv CH_4): HadGEM: 659, CESM2: 518, GFDL: 381, GISS: 364, MIROC 296, MMM: 480. These population-weighted responses for each model are slightly larger than the corresponding global average given that sunlight, water vapor, and halogens efficiently destroy ozone over the ocean [*Read et al.*, 2008] and net production tends to increase over populated regions with abundant precursor emissions.

In addition to global average results, Figure S2 also shows that the O_3 response to methane is spatially heterogeneous. While the spatial patterns are slightly different across each model, each model predicts that the largest anticipated changes are primarily centered over the Middle East and central, south, and east Asia. The patterns and magnitudes of calculated ozone changes in Figure S2 in response to methane are also consistent with the UNEP/CCAC Assessment, such that the UKESM model has the largest average O_3 response to changes in methane emissions (global average change: 383 pptv/100 ppbv CH_4), while the MIROC-CHASER has the smallest (global average change: 274 pptv/100 ppbv CH_4).

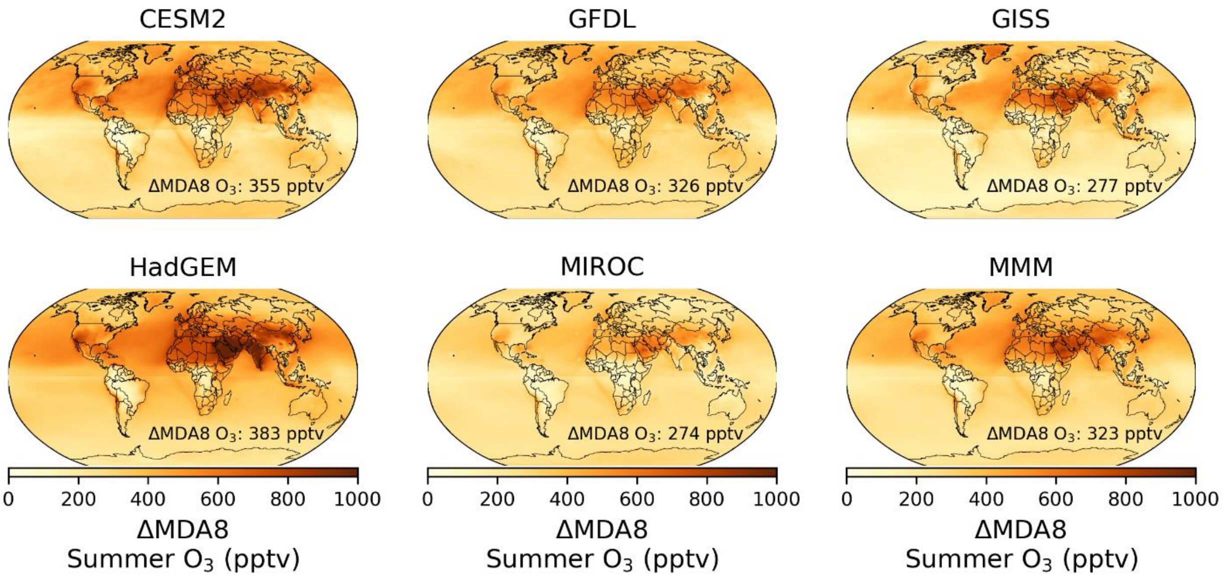

Figure S2. Calculated changes in summertime MDA8 ozone mixing ratios (in units of pptv) in 2020 for each model and the calculated multi-model mean (MMM), in response to a 100 ppbv methane emissions pulse in the same year. The global average change (no weighting) is provided in each panel.

Figures S1 shows that due to the lifetime of CH_4 and O_3, the global atmospheric abundance of both CH_4 and O_3 return to their initial 2020 levels well before the end of the century, such that integrating the impacts between 2020 and 2100 will capture the majority of climate damages resulting from this methane-ozone-health mechanism.

Text S3. Population & Mortality Characteristics

As described in the main text, projections of total population and background respiratory mortality rates are calculated for each country using a combination of data from the Resources for the Future-Socioeconomic Projections dataset (RFF-SP) [*Rennert et al.*, 2022b] and the International Futures Project (IFP) [*International Futures (IFs) modeling system*]. The public RFF-SP database contains 10,000 probabilistic projections of greenhouse gas emissions, total population, and gross-domestic product (GDP) for 184 countries from 2020-2300. As described in *Rennert et al.* [2021], total population data in the RFF-SP dataset are drawn from 1000 individual population projections from *Raftery and Ševčíková* [2023], which in part rely on projections of country-specific, age- and sex-stratified background mortality rates. We obtained this population and all-cause mortality dataset via personal communication from H. *Ševčíková*, which contains 1000 individual projections of population and mortality rates for 19 age bins (every five years from ages 0-4 through 90+) and 201 countries.

To derive respiratory-specific mortality rates, the 1000 individual country-specific all-cause rates are scaled by the ratio of projected respiratory-to-all-cause mortality – by age and country- from the International Futures Project [*International Futures (IFs) modeling system*]. The IFP dataset contains respiratory and all-cause mortality rates from 2000-2100, by sex, for 22 age bins and 186 countries. These age bins were merged into the same 19 bins as the RFF-SP data and the sex-stratified rates were used to find the population weighted average rates. For the 15 countries in the RFF data that are not

included in the IFP dataset (primarily small islands nations), respiratory-all-cause mortality ratios were assigned to those in the nearest geographical country. Figure S3 shows the resulting global projections of total population and calculated global respiratory mortality rate for the 1000 projections. Central results presented throughout the main text are derived using the average of these data (Figure S3, red line). Note that consistent with similar types of air quality impact studies, this study design does not account for the effects of respiratory deaths on the projected populations as the population projections are developed separately from the ozone-health modeling conducted here.

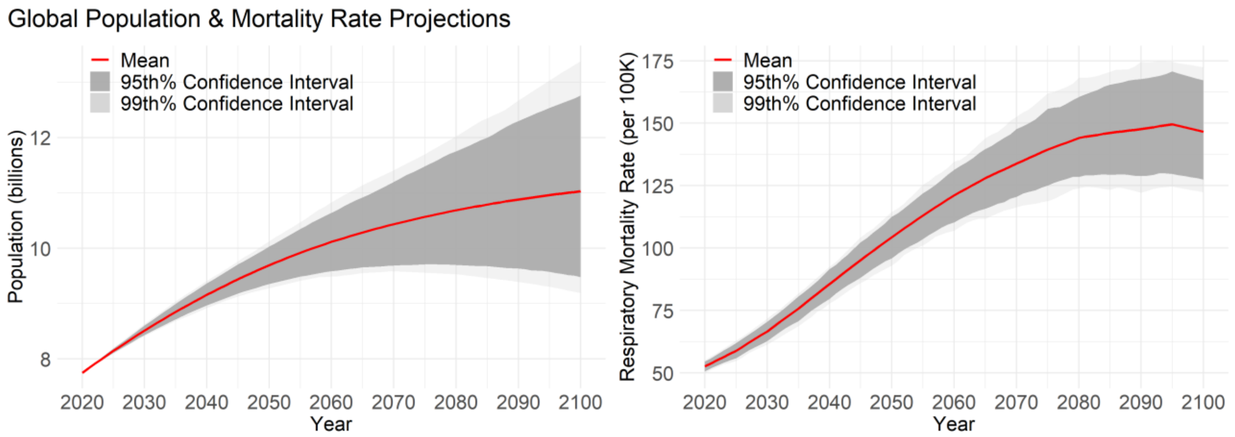

Figure S3. Projections of global population and respiratory mortality rates. Mean, 95th, and 99th percent confidence intervals are shown in red, gray, and light gray respectively. Global population data are aggregated across country, age, and sex. Global respiratory mortality rates are calculated as the aggregate of country-level respiratory mortality counts (= $\sum$country mortality rate × country population), divided by the global total population (aggregated across country, age, and sex).

Additional socioeconomic sensitivity tests presented in Section 3 of the main text are conducted with the full set of 10,000 public RFF-SP population and GDP data, paired with the corresponding background mortality projections using a crosswalk between the public RFF-SP trial number and the corresponding draw number from the 1000 population/mortality projections.

Text S4. BenMAP Mortality Estimates

As described in the main text, the new BenMAP cloud-based webtool was expanded in this work to cover the global region and leverage cloud computing resources. The BenMAP webtool used inputs of re-gridded 0.5° x 0.5° global maps of MDA8 O_3 (with and without 2020 CH_4 pulse perturbations) calculated for each of the 5 GCMs, as well as downscaled global grids of total population and baseline respiratory-related mortality rates. BenMAP then aggregates population and mortality data across the 0-99 age group and calculates the chronic respiratory-mortality attributable to the change in ozone exposure for each country following Eq. 1 in the main text. The code and documentation for an archived version of the BenMAP application is here: https://zenodo.org/record/7930887. Additional code used to run the global BenMAP webtool are available on the BenMAP github repository: https://github.com/BenMAPCE/BenCloudApp/tree/develop-global-ozone; https://github.com/BenMAPCE/BenCloudServer/tree/develop-global-ozone

This analysis focuses on respiratory-related mortality impacts on ages 0-99 years from long-term ozone exposure. This study does not quantitatively consider additional health endpoints from long-term exposure, such as from cardiovascular disease. This aligns with recent causality determinations published in the 2020 U.S. EPA Integrated Science Assessment for Ozone and Related Photochemical Oxidants: “Collectively, the body of evidence for long-term ozone exposure and cardiovascular effects is suggestive of, but insufficient to infer, a causal relationship” [U.S. EPA, 2020, pg. 4-64]. “Overall, the collective evidence is sufficient to conclude that a likely to be causal relationship exists between long-term ozone exposure and respiratory effects” [U.S. EPA, 2020, pg. 3-116]. As the detailed understanding of mechanistic health impacts from air pollution exposure is an active field of research, physical and monetized impacts associated with ozone exposure may require revisions as new information becomes available.

As this analysis is not a standard ‘burden’ analysis and is instead focused on estimating increases in mortality attributable to ozone from an additional pulse of methane emissions, we do not implement a theoretical minimum risk exposure level (TMREL). The 2019 GBD recently suggested a uniform distribution of the long-term ozone TMREL between 29.1 and 35.7 ppbv, based on the underlying studies [*GBD 2019 Risk Factor Collaborators*, 2020]. Implementing the median TMREL from this distribution (32.4 ppbv), following the approach of *Malashock et al.* [2022], would reduce the integrated total number of global ozone attributable respiratory-related deaths in this analysis by 3.1% (or 6,500 deaths).

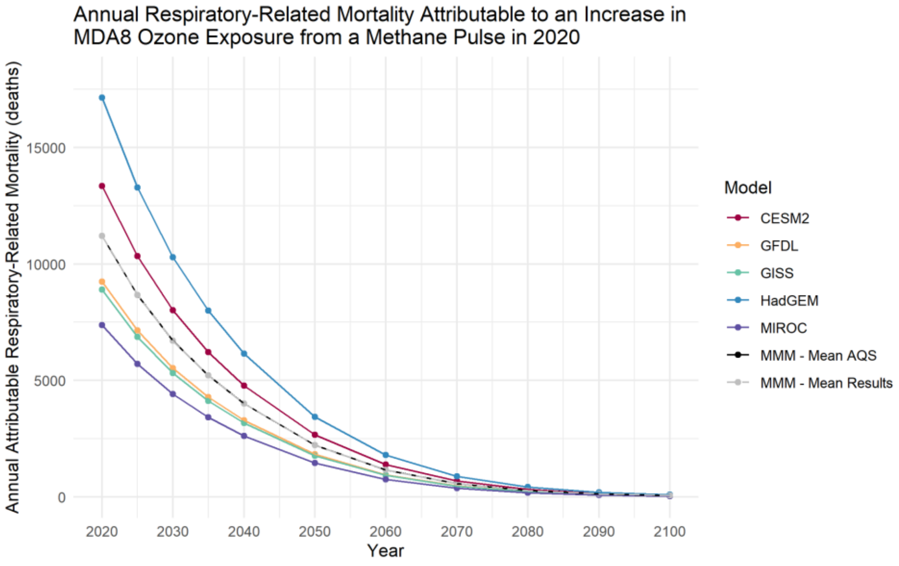

Figure S4. Timeseries of annual global methane-ozone attributable respiratory deaths, by GCM

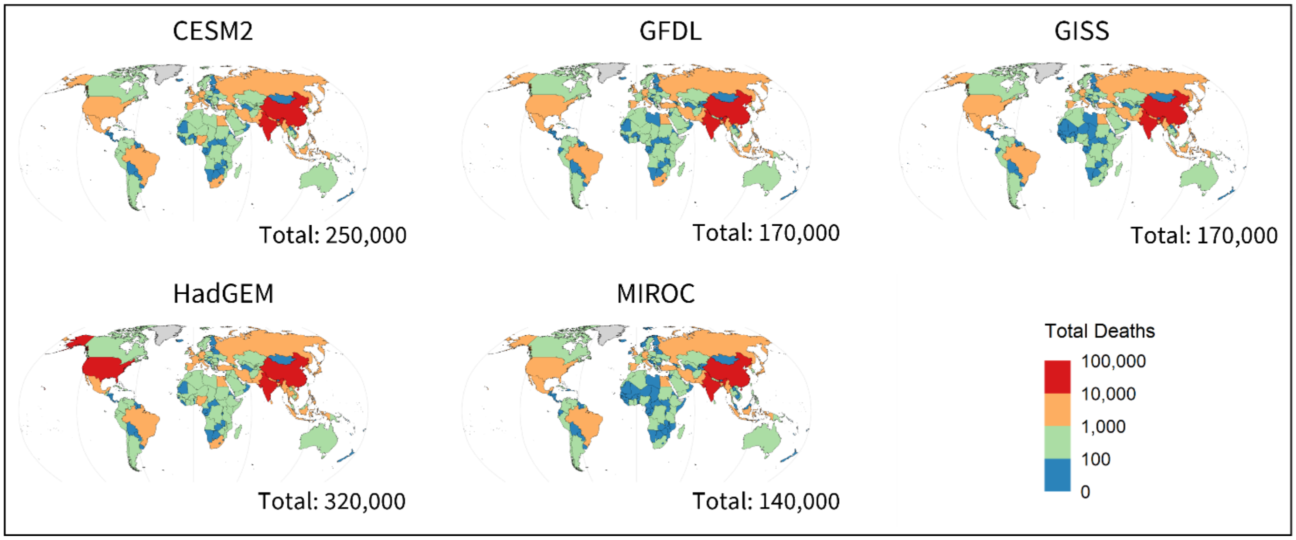

Figure S5. Maps of total integrated methane-ozone respiratory related attributable deaths, by country and model.

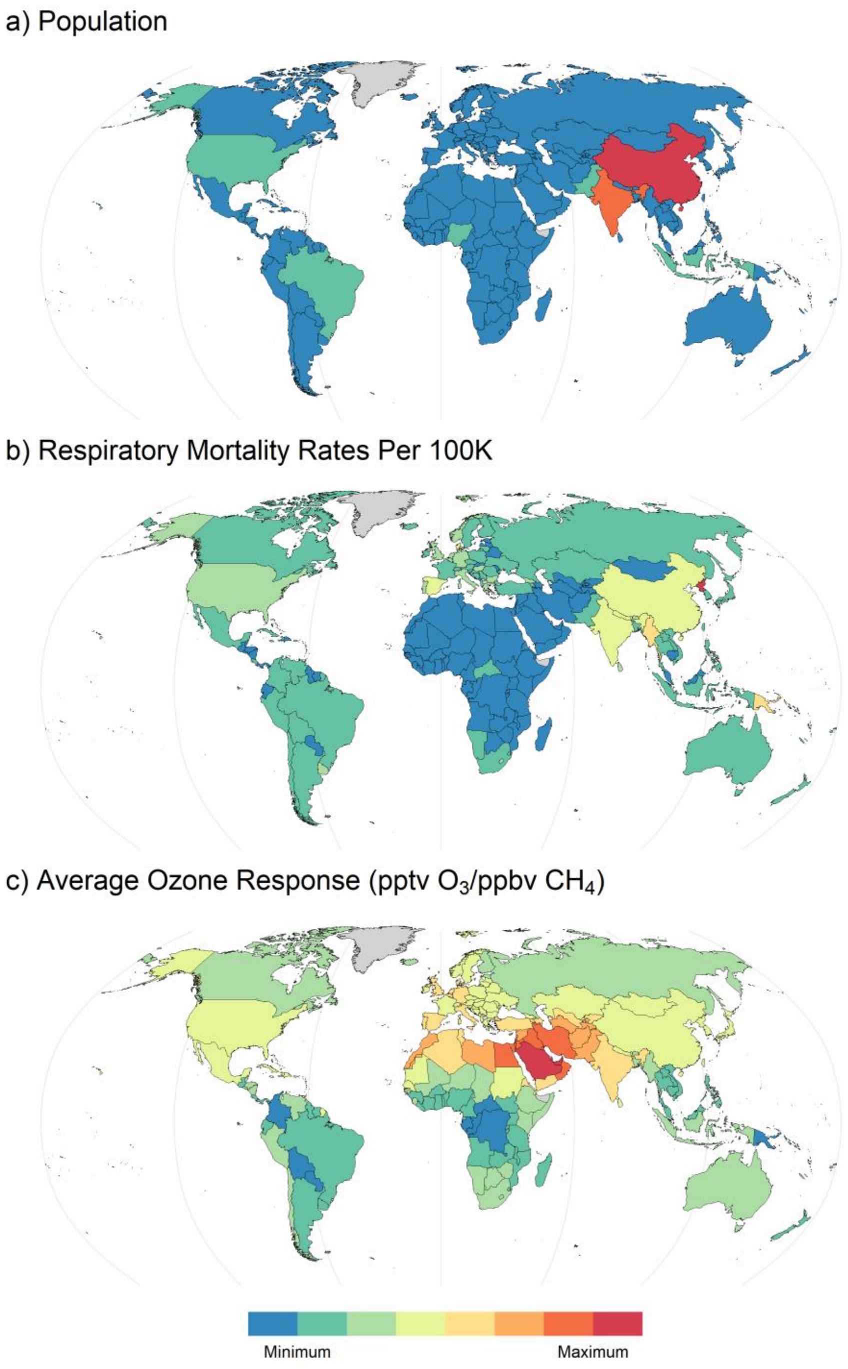

Figure S6. Snapshot of country differences in the year 2020 in a) population, b) background respiratory mortality rates per 100K, and c) modeled average ozone response to methane changes. The color scale ranges from the minimum to maximum value in each panel, by country.

Text S5. Monetization

The damages associated with increased respiratory-related deaths attributable to ozone formed from a marginal methane emissions pulse are monetized using country-specific VSL estimates. As described in Section S3, the full RFF population & mortality dataset from *Raftery and Ševčíková* [2023], contain data for 201 countries (Table S1), while the public version of the full 10,000 probabilistic emission, population, and GDP draws only contain data for 184 countries. For those 17 countries in the public RFF-SP dataset that were not included in the underlying population dataset (primarily small island nations), VSL estimates are assigned to those calculated at the broader region level (Table S1).

Annual mortality counts for each country and GCM through the end of the century are then discounted back to 2020 U.S. dollars using multiple discounting approaches. The first uses constant discount factors of 2.0% and 3.0%. The second approach follows recent literature and the 2017 Council of Economic Advisors Issue Brief [CEA, 2017] to apply a time-varying Ramsey discounting approach (Eq. 3), calibrated to near-term discount rates of 1.5%, 2.0% (presented in the main text), 2.5%, and 3.0% [*Rennert et al.*, 2022a]. For these rates, the values for ρ= 0.01%, 0.2%, 0.5%, 0.8% and η= 1.02, 1.24, 1.42, 1.57, respectively.

Consistent with *Rennert et al.* [2022a], we calculate the stochastic discount rate to discount future marginal mortality-related damages from the methane-ozone-health mechanism. The stochastic discount factor can be written in terms of relative consumption levels for each year (*t*) and country (*c*), following Eq. S3.

$$Stochastic\ Ramsey\ Discount\ Factor_{c,t} = \frac{1}{(1+\rho)^{t-2020}}\left(\frac{c_t}{c_{2020}}\right)^{-\eta} \qquad \text{(Eq. S3)}$$

Where c_t in this work is the country level per capita consumption in year t and η is transformed by η = exp(η)-1. In this analysis, we use country specific VSL estimates, income growth, damages, and discounting. This differs from the SC-CH_4 calculation of Ramsey discount rates, which uses country-specific VSL estimates, but takes c_t as world average consumption rate [*Rennert et al.*, 2022a]. Applying a global average consumption rate to discount the methane-ozone damages in this analysis increases the global NPV by ~7%. The stochastic discount factors for each year and country are then multiplied by the marginal damages and aggregated over time into a single present value.

$$\text{NPV}_{c,t} = \sum_{t=2020}^{t=2100} \text{SDF}_{c,t} \times \text{Marginal Damages}_{c,t} \qquad \text{(Eq. S4)}$$

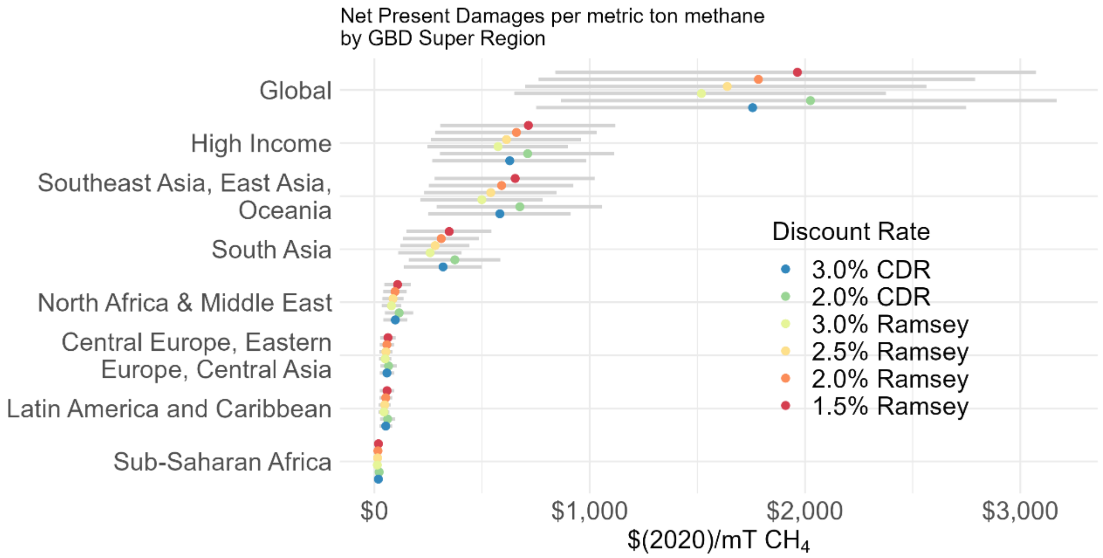

Figure S7. Net Present Value (NPV) per ton of methane emitted in 2020, as a function of region and discount factor.

Table S1. Country names and regions included in this analysis. Country names and groups are consistent with those in the Global Burden of Disease project. The relative ranking and NPVs (2020$/mT CH_4; 2% Ramsey discount rate) are listed for the largest 20 countries.

Central Europe, Eastern Europe, Central Asia
Albania
Armenia
Azerbaijan
Belarus
Bosnia and Herzegovina
Bulgaria
Croatia
Czech Republic
Estonia
Georgia
Hungary
Kazakhstan
Kyrgyzstan
Latvia
Lithuania
Moldova
Mongolia
Montenegro
North Macedonia
Poland
Romania
Russian Federation (12; $20/mT)
Serbia
Slovakia
Slovenia
Tajikistan
Turkmenistan
Ukraine
Uzbekistan

High Income
Argentina
Aruba
Australia
Austria
Belgium
Brunei Darussalam
Canada (15; 20/mT)
Channel Islands
Chile
Curacao
Cyprus
Denmark
Finland
France (11; $20/mT)
French Guiana
French Polynesia
Germany (5; $60/mT)
Greece
Guadeloupe
Iceland
Ireland
Israel
Italy (9; $30/mT)
Japan (4; $60/mT)
Luxembourg
Malta
Martinique
Mayotte
Netherlands (20; $20/mT)
New Caledonia
New Zealand
Norway
Portugal
Reunion
San Marino
Singapore
South Korea (14; $20/mT)
Spain (7; $40/mT)
Sweden
Switzerland
United Kingdom (6; $50/mT)
United States (3; $270/mT)
Uruguay

Latin America and Caribbean
Antigua and Barbuda
Bahamas
Barbados
Belize
Bolivia
Brazil (13; $20/mT)
Colombia
Costa Rica
Cuba
Dominican Republic
Ecuador
El Salvador
Grenada
Guatemala

Guyana
Haiti
Honduras
Jamacia
Mexico (16; $20/mT)
Nicaragua
Panama
Paraguay
Peru
Puerto Rico
Sant Lucia
Sant Vincent and the Grenadines
Suriname
Trinidad and Tobago
US Virgin Islands
Venezuela

North Africa & Middle East

Afghanistan
Algeria
Bahrain
Egypt
Iran
Iraq
Jordan
Kuwait
Lebanon
Libya
Morocco
Oman
Palestinian Territory
Qatar
Saudi Arabia (19; $20/mT)
Sudan
Syria
Tunisia
Turkey (8; $30/mT)
United Arab Emirates
Western Sahara
Yemen

South Asia

Bangladesh
Bhutan
India (2; $290/mT)
Nepal
Pakistan (18; $20/mT)

Southeast Asia, East Asia, Oceania

Cambodia
China (1; $490/mT)
Hong Kong
Macao
Taiwan
Fiji
Guam
Indonesia (10; $30/mT)
Kiribati
Laos
Malaysia
Maldives
Mauritius
Micronesia
Myanmar
North Korea (17; $20/mT)
Papua New Guinea
Philippines
Samoa
Seychelles
Solomon Islands
Sri Lanka
Thailand
Timor-Leste
Tonga
Vanuatu
Vietnam

Western, Sub-Saharan Africa

Angola
Benin
Botswana
Burkina Faso
Burundi
Cabo Verde
Cameroon
Central African Republic
Chad
Comoros
Congo
Democratic Republic of the Congo
Cote d'Ivoire
Djibouti
Equatorial Guinea
Eritrea
Eswatini
Ethiopia
Gabon
Gambia
Ghana
Guinea
Guinea-Bissau
Kenya
Lesotho
Liberia
Madagascar
Malawi
Mali
Mauritania
Mozambique
Namibia
Niger
Nigeria
Rwanda
Sao Tome and Principe
Senegal
Sierra Leone
Somalia
South Africa
South Sudan
Tanzania
Togo
Uganda
Zambia
Zimbabwe

We also test the sensitivity of these monetized results to a 20-year mortality cessation lag (Table S2). This lag accounts for the time duration between initial exposure and death and has historically only been applied in U.S. EPA analyses for deaths resulting from particulate matter exposure. Mortality resulting from long-term ozone exposure may result in similar health outcomes as particulate matter, including chronic respiratory disease and lung cancer. To implement the cessation lag, we distribute the annual mortality counts from the BenMAP webtool, using the lags in Table S2. The cessation-adjusted mortality counts are then monetized and discounted using the same approach as described above. While most ozone-attributable deaths are estimated to occur in 2020 (Figure S4), implementation of the cessation lag distributes these to later years resulting in net present damages of $1700/ton CH_4 (2% Ramsey), which is roughly a 2.5% reduction in damages compared to the central estimate presented in the main text.

Table S2. 20-year mortality cessation lag

Year(s)	Fraction of deaths attributable to initial O_3 exposure that occur in each subsequent year
0	30%
1-4	12.5%
6-19	1.3%

Text S6. Methane-Ozone Mortality Model – Reduced Form Tool

As described in the main text, due to the computational requirements to run the global cloud-based BenMAP tool under multiple future scenarios, we additionally develop an R-based reduced form tool to test the sensitivities of the global NPV to changes in socioeconomic and precursor emission projections. The reduced form tool leverages the near linear relationships between changes in long-term O_3 exposure levels and population and background mortality characteristics with changes in attributable mortality.

To evaluate the reduced form tool, we run a series of select additional BenMAP simulations for specific individual RFF-SP projections. Running the reduced form tool for the same projection number reveals that the global respiratory-related mortality estimates from the reduced form tool are within 0.5% of the BenMAP calculated results for all tested simulations. Individual year- and projection-specific estimates may be greater. The country-level mortality counts from the reduced form tool and from the original BenMAP runs are then monetized and discounted using the same methodology. Therefore, the BenMAP derived mortality results provide the most accurate respiratory-related mortality estimates for a specific future scenario, but the development of the reduced form approach allows us to quickly test additional sensitivities of the NPV to a large range of future conditions.

The reduced form tool has also been designed to facilitate the calculation of NPV (i.e., SC-CH_4) associated with a custom methane emissions pulse under any socioeconomic scenario. The model currently allows users to specify parameters such as the methane emission pulse size, methane perturbation lifetime, pulse year, the cessation lag (if implementation is selected), the income elasticity, and value of a statistical life. Other inputs include 2020-2100 projections of country-level population, background respiratory related mortality, and GDP. As population and background mortality are inherently linked, the tool is currently equipped to run any of the 10,000 probabilistic public RFF-SP scenarios [*Rennert et al.*, 2022b]. Lastly, the user may also choose to input a projection of country-level NO_x emissions (in megatons/year) or a single NO_x emission scaling factor. If a scaling factor is chosen, NO_x levels in each country are held constant over time at the 2015 emissions levels used in the original UNEP/CCAC simulations, multiplied by the scaling factor (e.g., new NO_x = NO_x scalar * original NO_x). In either case, the timeseries of NO_x emissions are used in the tool to calculate the change in methane-O_3 production efficiency, following the ΔO_3 response/NO_x emissions relationships in Eq. 6 in the main text, derived as part of the original UNEP/CCAC Assessment [UNEP/CCAC, 2021].

References

Council of Economic Advisers (2017), Discounting for Public Policy: Theory and Recent Evidence on the Merits of Updating the Discount Rate,

https://obamawhitehouse.archives.gov/sites/default/files/page/files/201701_cea_discounting_issue_brief.pdf

GBD 2019 Risk Factor Collaborators (2020), Global burden of 87 risk factors in 204 countries and territories, 1990–2019: a systematic analysis for the Global Burden of Disease Study 2019, *The Lancet*, *396*(10258), 1223-1249, doi:https://doi.org/10.1016/S0140-6736(20)30752-2.

International Futures (IFs) modeling system Version 7.88, Pardee Center for International Futures, Josef Korbel School of International Studies, University of Denver, Denver, CO, https://korbel.du.edu/pardee/international-futures-platform/download-ifs.

Malashock, D. A., M. N. DeLang, J. S. Becker, M. L. Serre, J. J. West, K.-L. Chang, O. R. Cooper, and S. C. Anenberg (2022), Estimates of ozone concentrations and attributable mortality in urban, peri-urban and rural areas worldwide in 2019, *Environ. Res. Lett.*, *17*(5), 054023, doi:10.1088/1748-9326/ac66f3.

Prather, M. J., C. D. Holmes, and J. Hsu (2012), Reactive greenhouse gas scenarios: Systematic exploration of uncertainties and the role of atmospheric chemistry, *Geophys. Res. Lett.*, *39*(9), doi:https://doi.org/10.1029/2012GL051440.

Raftery, A. E., and H. Ševčíková (2023), Probabilistic population forecasting: Short to very long-term, *International Journal of Forecasting*, *39*(1), 73-97, doi:https://doi.org/10.1016/j.ijforecast.2021.09.001.

Read, K. A., et al. (2008), Extensive halogen-mediated ozone destruction over the tropical Atlantic Ocean, *Nature*, *453*(7199), 1232-1235, doi:10.1038/nature07035.

Rennert, K., et al. (2022a), Comprehensive evidence implies a higher social cost of CO2, *Nature*, *610*(7933), 687-692, doi:10.1038/s41586-022-05224-9.

Rennert, K., et al. (2021), The Social Cost of Carbon: Advances in Long-Term Probabilistic Projections of Population, GDP, Emissions, and Discount Rates, *Brookings Papers on Economic Activity*, 223-275.

Rennert, K., et al. (2022b), The Social Cost of Carbon: Advances in Long-Term Probabilistic Projections of Population, GDP, Emissions, and Discount Rates, edited, doi:10.5281/zenodo.5898729.

Szopa, S., et al. (2021), Short-Lived Climate Forcers. In: Climate Change 2021: The Physical Science Basis. Contribution of Working Group I to the Sixth Assessment Report of the Intergovernmental Panel on Climate Change., pp. 817-922 pp, Cambridge University Press, Cambridge, United Kingdom and New York, NY, USA., doi:10.1017/9781009157896.008.

U.S. Environmental Protection Agency (2020), Integrated Science Assessment for Ozone and Related Photochemical Oxidants, https://cfpub.epa.gov/ncea/isa/recordisplay.cfm?deid=348522.

United Nations Environment Programme and Climate and Clean Air Coalition (2021), *Global Methane Assessment: Benefits and Costs of Mitigating Methane Emissions.*, United Nations Environment Programme, Nairobi.
